# Cement floor tiles based on waste ceramic and waste tires for EMI shielding

**DOI:** 10.1038/s41598-026-48682-1

**Published:** 2026-04-30

**Authors:** Rawya M. Ramadan, Emad S. Shafik, Nadia F. Youssef, Salwa L. Abd-El Messieh, Azza A. Ward

**Affiliations:** 1 Microwave Physics and Dielectric Department, National Research Centre (NRC), Giza, Egypt; 2 Polymer and Pigments Department, National Research Centre (NRC), Giza, Egypt; 3 Raw Building Materials Technology and Processing Research Institute, Housing and Building National Research Centre (HBRC), Dokki, Giza, Egypt

**Keywords:** Demolition waste ceramic tiles, Waste iron powder (WIP), Cement floor tiles, Water absorption compression strength, Flexural strength, Electrical conductivity, EMI shielding, Engineering, Environmental sciences, Materials science

## Abstract

This study explores the valorization of ceramic waste (CW) and waste tire particles in the development of eco-friendly cementitious tiles for outdoor roof shielding applications. CW, sourced from industrial byproducts and demolition debris, offers promising hydraulic properties and cost-effectiveness. Two waste samples, collected during the renovation of sanitary facilities in an aged building and waste iron powder (WIP) were incorporated into cement formulations comprising Portland cement, fine aggregates, water, and recycled materials. The waste components were characterized via particle size distribution analyses which was found in the order of 34.15 μm for waste wall ceramic while it was 49.06 μm for waste floor one. The chemical composition analysis using X-ray fluorescence (XRF) was measured. The bulk density after a cure period of 28 days, water absorption was also evaluated. The compressive strength and flexural strength data revealed enhancement by the addition of WIP particles. This enhancement is attributed to the strong interfacial bonding between WIP particles and the cementitious matrix. Powder X-ray diffraction was used to measure crystalline phase composition. The results demonstrate that ceramic and rubber wastes reduce density and increase water absorption due to enhanced porosity, while the inclusion of WIP significantly improves matrix densification, mechanical strength, and electrical conductivity. Composites containing 10 wt% WIP exhibited optimal performance, achieving enhanced compressive and flexural strengths. Electrical conductivity measurements revealed values ranging from 10^− 13^ to 10^− 11^ S/cm, aligning with the requirements for antistatic applications. Consequently, the tiles are recommended for use as antistatic roof shielding materials. Besides, Electromagnetic interference (EMI) shielding tests demonstrated that samples incorporating a metal mesh achieved attenuation levels exceeding 20 dB, effectively blocking over 99% of incident electromagnetic waves. Further enhancement was observed with the addition of waste conducting particles (WIP), suggesting that composites integrating both WIP and metal mesh can achieve EMI shielding efficiencies up to 99.999%, making them suitable for industrial and commercial applications demanding high-performance shielding. The developed tiles comply with Egyptian and European standards for external cement tiles, demonstrating their suitability for sustainable construction applications, particularly for roofing and flooring in environments exposed to electromagnetic pollution. This work highlights an effective pathway for converting multiple waste streams into high-value, multifunctional building materials.

## Introduction

In recent decades, rapid industrialization and urban growth have markedly augmented the production of various waste streams, especially those resulting from construction and demolition (C&D) activities. The accumulation of such waste poses a continual environmental challenge, frequently requiring the creation of remote landfills for disposal management. In response, integrated waste management techniques and recycling activities have become essential elements of sustainable development frameworks. In Egypt, this necessity has been formalized with the creation of the Waste Management Regulatory Authority (WMRA), responsible for supervising national waste administration in accordance with the nation’s Vision 2030 sustainability framework^1,2^.

Ceramic wall and floor tiles constitute a significant portion of construction and demolition trash due to their non-biodegradable characteristics and restricted opportunities for on-site reuse. Their disposal in landfills exacerbates environmental damage and depletes resources. Recent studies have investigated the utilization of waste ceramic tiles (WCTs) as partial substitutes for fine aggregates or cement in non-structural concrete applications, highlighting advantages such as diminished CO₂ emissions and improved durability attributed to their elevated alumina and silica content^3–6^. The use of waste and by-product materials to substitute for concrete’s ingredients is one facet of many researches as a result; less waste from residential and commercial sources is dumped into the environment^7^. A comprehensive review published recently, edited by many researchers from many countries in Asia and Europe, concentrated mainly about utilization of ceramic waste as a major constituent in cement and concrete through recycling from many sources as a required sustainability either from industry or construction and demolition debris. The recommends finally was that future studies should concentrate on synergistic effects of other industrial wastes when combined with WCTs, with special care about the properties of the end product and its durability^8^.

Two different demolition wastes: terracotta roof tiles and sanitary porcelain were added as a substitute for traditional calcined clays in blended cement. As a recommendation to produce ecofriendly cement, both economic and environmental assessment become necessary regarding the purification of porcelain waste, depending on the source and preparation processes in situ^9^. A group of researchers from Brazil^10^ replaced Portland cement by ceramic tiles demolition waste. The ceramic waste showed better performance especially with the compressive strength with 5% over. Very interesting discussions were found by many researchers about what is the ultimate percentage of replacing both sand and other coarser aggregates by WCTs in mortars and concrete to be complying with standards and be suitable for use in its proper purposes^8,11^.

The production of paving units for both face and backing layers utilizing waste construction were investigated. The conclusion was that the use of fine crushed waste ceramics showed better performance than using coarse one at the same replacement percentages of natural aggregates^12,13^. Recent advances emphasize the valorization of industrial and construction wastes (e.g., recycled concrete powder, brick waste, volcanic pumice) as alumino-silicate precursors in eco-friendly geopolymer systems with enhanced mechanical performance and reduced environmental impact^14^. Furthermore, advanced clay-based geopolymer research elucidates key structural and processing parameters that influence strength and sustainability in green binder materials^15^. The frequency of tire replacements has increased in tandem with the global car population growth^16^, which has resulted in a steady rise in waste tire (WT) production in recent years^17^. Every year, an estimated about 1.5 billion tires are thought to reach the end of their useful lives^18^. WT are non-biodegradable supplies, and keeping them outdoors for an extended period of time not only uses a lot of land but also seriously pollutes the environment^19^. Worldwide, the high-value use of WT has grown to be a serious ecological problem^20^. Therefore, a global challenge is to find a non-hazardous and cost-effective way to dispose of WT^21^.

The process of shielding any material from the destructive effects of stray radiations is known as electromagnetic interference (EMI) shielding. In the Composite that functions in EMI shielding and MA shielding, properties such as ductility and tensile properties, are in high demand^22–24^. Researchers have been committed to creating MA materials and high-efficiency EMI shielding in order to address these issues. Numerous materials, such as metals and their oxides^25,26^, carbon-based materials^27,28^, and intrinsic conductive polymer materials^29,30^, have found widespread use in EMI shielding and MA in recent decades. It is anticipated that the primary focus of future industry development will be on leveraging the synergistic effect of composite materials to achieve a variety of applications^31^. The EMI material must have high electrical conductivity, relative permittivity, magnetic permeability, and a large internal surface area in order to have good shielding properties^31^.

This study aims to develop and establish innovative cementations’ tiles for outdoor use by integrating ceramic waste from demolition, recycled tire rubber, and by-products from the iron sector. The main goal is to convert these common, environmentally detrimental wastes into high-performance, eco-efficient construction materials with inherent EMI shielding properties. Also, this work examines the physicochemical, mechanical, and electromagnetic properties of the resultant composites, assisting the overarching goal of sustainable material innovation in the built environment.

## Experimental work

### Materials

#### Demolition wastes of ceramic wall and floor tiles

Two samples of wastes were obtained during a demolition process of renewing bathrooms and kitchens in an old building. They are expected to be aged for not less than 20 years. The ceramic material is expected to be mixed with old aged mortars. Being solid fractured tiles, they were ground in the laboratory, through a crusher then a mill to get it ground to the very fine size of 75 micron.

#### Waste rubber

**Fig. 1:**
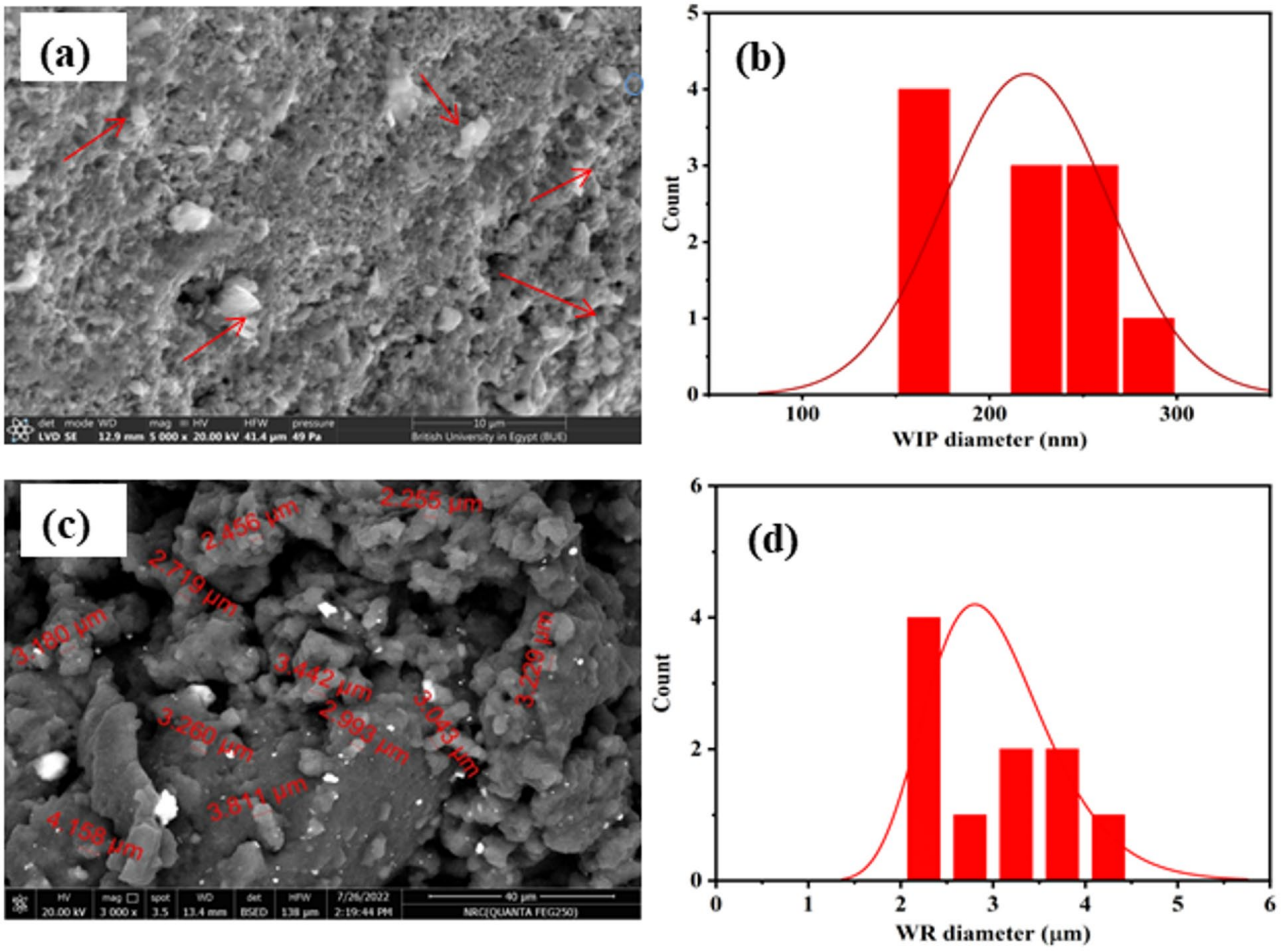
(**a**) and (**c**) SEM images of WIP and WR particles, whereas (**b**) and (**d**) are histogram showing mean size of WIP is 219.6 nm and, mean size of WCP is 258.7 nm mean size of WR is 3.12 μm.

The waste tire powder was obtained from locally used vehicle tires in Cairo, Egypt, Cairo, Egypt. The crumb rubber had a specific gravity of 0.98, bulk density of 0.55 t/m^3^ and mean particle size 3.12 μm Fig. 1.

#### Waste iron powder WIP

Waste Iron powder (WIP) is an industrial byproduct produced locally from mechanical processes including grinding, cutting, and milling of completed iron products. The mean particle size of WIP is 219.6 nm Fig. 1. Additional details regarding the characterization of WIP are available in earlier study^32^.

### Samples preparation

The samples used in this study are subdivided into three different groups and their mixing proportions are listed in Tables 1, 2 and 3. The codes AG1, AG2, AG3, AG4, and AG5, which correspond to the overall percentage of all waste by weight 0, 10, 20, 30, and 40%, were assigned to the samples of the first group I (Table (1): indicating the formulation of control sample without waste iron powder (WIP). The second group II (Table 2), which represents cement mortar with incorporated WIP, was coded as AG2, AG21, AG22, AG23, and AG24, respectively. AG2 is the optimum of Group I and serves as the group’s control (0%); the other samples, AG21, and AG24 correspond to the overall percentage of WIP by weight of 2.5, 5, 7.5, and 10%.Additionally, samples of the third group III (Table 3), which represent cement mortar with included WIP, were coded as AG2, AG31, AG32, AG33, and AG34, which correspond to the total percentage of WIP by weight of 2.5, 5, 7.5, and 10% with metal mesh.

**Fig. 2:**
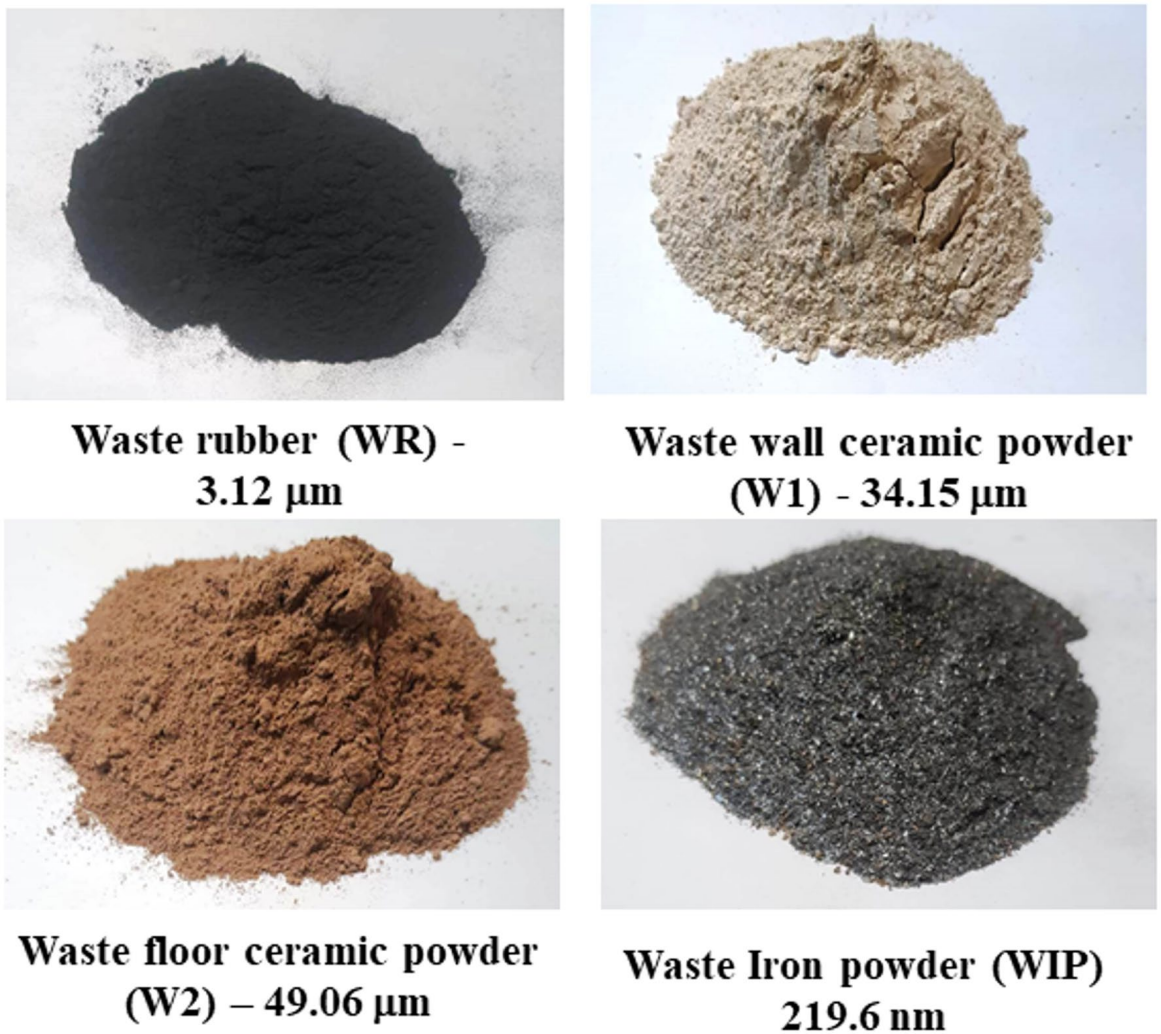
Images of the four different waste powders used in this study with their mean particle size.

The constituents employed in formulating the cement mixtures for this investigation encompassed cement, fine aggregate (sand), water, and wastes. The cement utilized was Ordinary Portland Cement EN 197-1-CEM152.5 N, in accordance with the certificate of conformity CE – 0770 – CPD – C02/23. The fine aggregate utilized was natural sand with a fineness modulus of 3.31. The investigation was conducted on the use of waste materials (fine ceramic waste wall/floor (W1/W2), waste rubber WR, and waste iron powder (WIP)) as a potential substitute for natural sand as illustrated in Fig. 2.

**Fig. 3:**
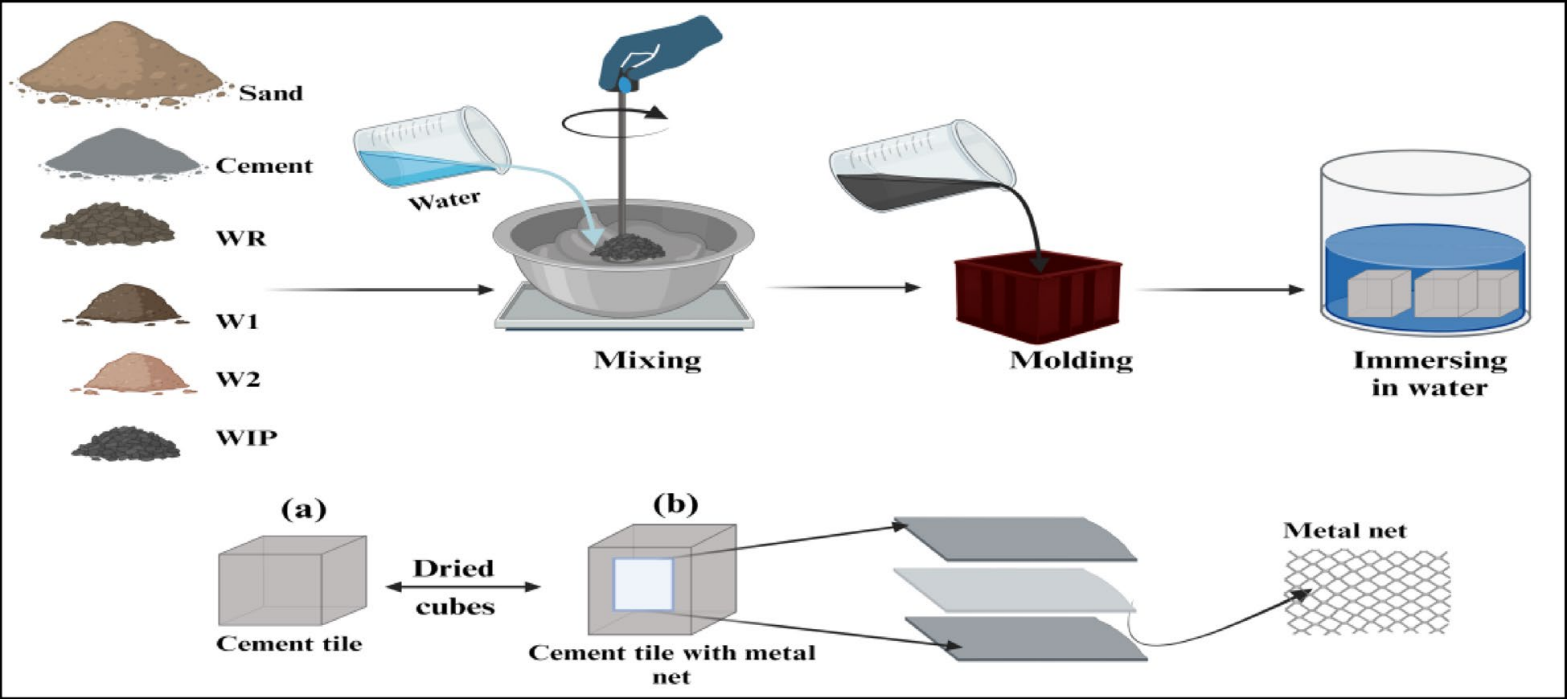
Schematic representation of preparation steps for cement tile.

The concrete samples were prepared by hand mixing (see Fig. 3); the mixing time was about 3–4 min. The resulting concrete samples were tested at intervals of 7, 14, and 28 days. For the compression test, cube specimens of 50 × 50 × 50 mm were created, whereas specimens measuring 10 × 4 × 80 mm were constructed for the flexure strength test (7, 14, and 28 days).

**Table 1:** Mixing proportions of the samples for Group No. I.

Group (I)	Total Percentage of all waste by weight	Mixing proportions (grams/one cube)					
		Cement	Water	Sand	WR	W1	W2
AG1	0	185	93	555	0	0	0
AG2	10	185	93	499.5	27.5	13.9	13.9
AG3	20	185	93	444	55.5	27.75	27.75
AG4	30	185	93	388.5	83.5	41.75	41.75
AG5	40	185	93	333	111	55.5	55.5

**Table 2:** Mixing proportions of samples for Group No. II.

Group (II)	Total Percentage of all waste by weight	Mixing proportions (grams/one cube)						
		Cement	Water	Sand	WR	W1	W2	WIP
AG2	0	185	93	555	0	0	0	0
AG21	2.5	185	93	499.5	27.5	13.9	13.9	13.9
AG22	5	185	93	499.5	27.5	13.9	13.9	27.75
AG23	7.5	185	93	499.5	27.5	13.9	13.9	41.63
AG24	10	185	93	499.5	27.5	13.9	13.9	55.5

**Table 3:** Mixing proportions of samples with metal net for Group No. III.

Group (III)	Total Percentage of all waste by weight	Mixing proportions (grams/one cube)						
		Cement	Water	Sand	WR	W1	W2	WIP
AG2	0	185	93	555	0	0	0	0
AG31	2.5	185	93	499.5	27.5	13.9	13.9	13.9
AG32	5	185	93	499.5	27.5	13.9	13.9	27.75
AG33	7.5	185	93	499.5	27.5	13.9	13.9	41.63
AG34	10	185	93	499.5	27.5	13.9	13.9	55.5

### Characterization techniques

#### Bulk density

The density of specimen was determined according to ASTM C642-97. The mean result was calculated from three samples, with an experimental error of 3%.

#### Water absorption

The water absorption of the samples was measured in accordance with the ASTM C642-97 standard^33^.The water absorption measurement process involved, immersing the samples in distilled water at (30 ± 5 °C) for 28- days. Then the samples were dried in an oven at a temperature range of 100–110 °C for duration of 24 h. Afterward, the samples were allowed to cool to room temperature and their weight was recorded (referred to as the “dry weight”). The calculation of water absorption was performed using the following equation;

$$\:Water\:absorption\:\left(\%\right)=\frac{W2-W1}{W1}\times100$$

where W1 is the mass of oven-dried specimen in air (gm), and W2 is the mass of dry specimen in air after immersion (gm). Three samples were measured for each composite and the mean value was taken, the experimental error was 3%.

### Compression strength test

**Fig. 4:**
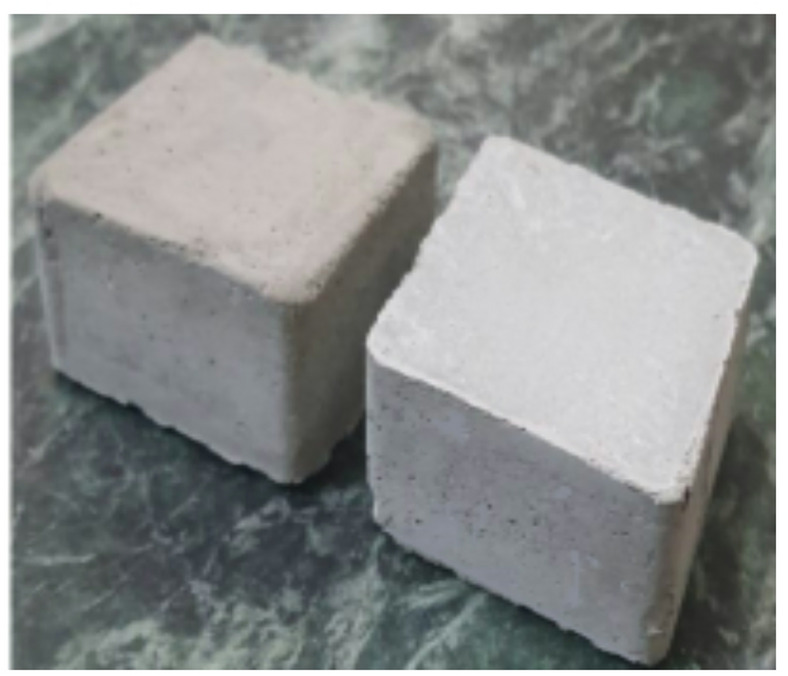
Concrete cubes for compression strength test.

For compression Experiment concrete cubes as in Fig. 3 are used. Three concrete cubes measuring 50 × 50 × 50 mm in size were made for each of the sample formulations listed in Tables 1, 2 and 3 containing different waste ratios, in addition to one cube utilizing regular concrete aggregates. All measurements were performed following the standardized methodology outlined in ASTM C579. There were a total of 45 samples. Figure 4 displays the cubes utilized in the compression test. The formula below was utilized to compute the compressive strength, measured in N/mm^2^ or MPa;

$$\:Compression\:strength=\frac{Crushing\:\:load\:\left(N\right)}{Crosssection\:\:area{(mm}^{2})}$$

### Flexural strength Test

**Fig. 5:**
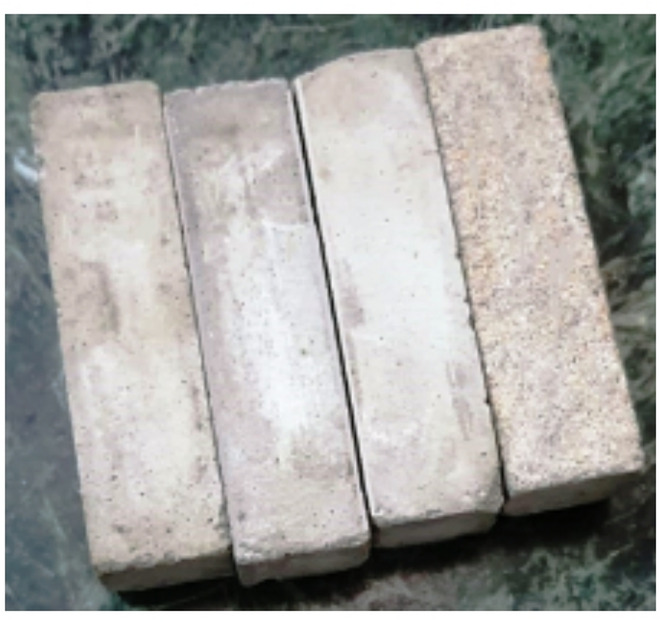
Concrete prism for flexural strength test.

Three point loading prism specimens for the flexural test of dimensions 25 × 30 × 150 mm prism specimens were used for the flexural strength test (see Fig. 5) according to ASTM C580.The calculation of the obtained flexural strength followed to the formula;

$$\:Flexural\:strength\left(\sigma\:\right)=\frac{FL}{b{d}^{2}}$$

*F* is the load (force) at the fracture point; *L* is the length of the support (outer) span, *b* is width and *d* is thickness, respectively.

### Particle size analysis

All ceramic wastes fineness was investigated through Laser Scattering Particle Size Distribution Analyzer apparatus (Horiba LA950) according to ASTM E3340-22. The measurements were volume distribution base.

### X-ray fluorescence technique (XRF)

Chemical analysis was carried out using Axios (PW4400) WD–XRF Sequential Spectrometer (Panalytical, The Netherlands), using (Rubidium) Rbka radiation tube at 50 kV and 50 mA.

### X-ray diffraction (XRD)

XRD data of the compounds were collected at ambient conditions on an Empyrean diffractometer by Panalytical (Almelo, The Netherlands), and filtered CuKα radiation, tube operated at 30 mA and 45 kV, and using a Ni filter to eliminate Kβ.

### Electrical conductivity measurements

The surface resistivity ρ was measured by Super MegOhm Meter Hioki SM7110 (Hioki, Japan). The samples were placed in shielding box type Hioki SME3811 with outer and inner electrode diameters 24 mm and 19 mm respectively. This shield box is guarded and tuned for applying DC potential up to 1000 V. The Super MegOhm Meter Hioki SM7110 is capable of measuring small current up to 1pA peco Amperes in resolution of 1fA femtoAmeres. From which the electrical conductivity σ was calculated. The electrical conductivity σ was calculated from the measured resistivity ρ according to the well-known relation σ = 1/ρ. The error in the measurement of ρ was 2%.

### EMI SE measurements

Within the X-band frequency range of 8–12 GHz, the EMI SE of neat composites was assessed. The test was performed using a sweep oscillator (HP 8350B, Hewlett Packard, Agilent Technologies, Melrose, MA, USA) connected to a Scalar Network Analyzer (HP 8757 C, Hewlett Packard, Agilent Technologies, Melrose, MA, USA). The test samples had a thickness of 1.3 mm. EMI SE was measured using three samples and then the average is taken. The error in the measurement was 3%.

## Results and discussion

### Particle size distribution

**Fig. 6:**
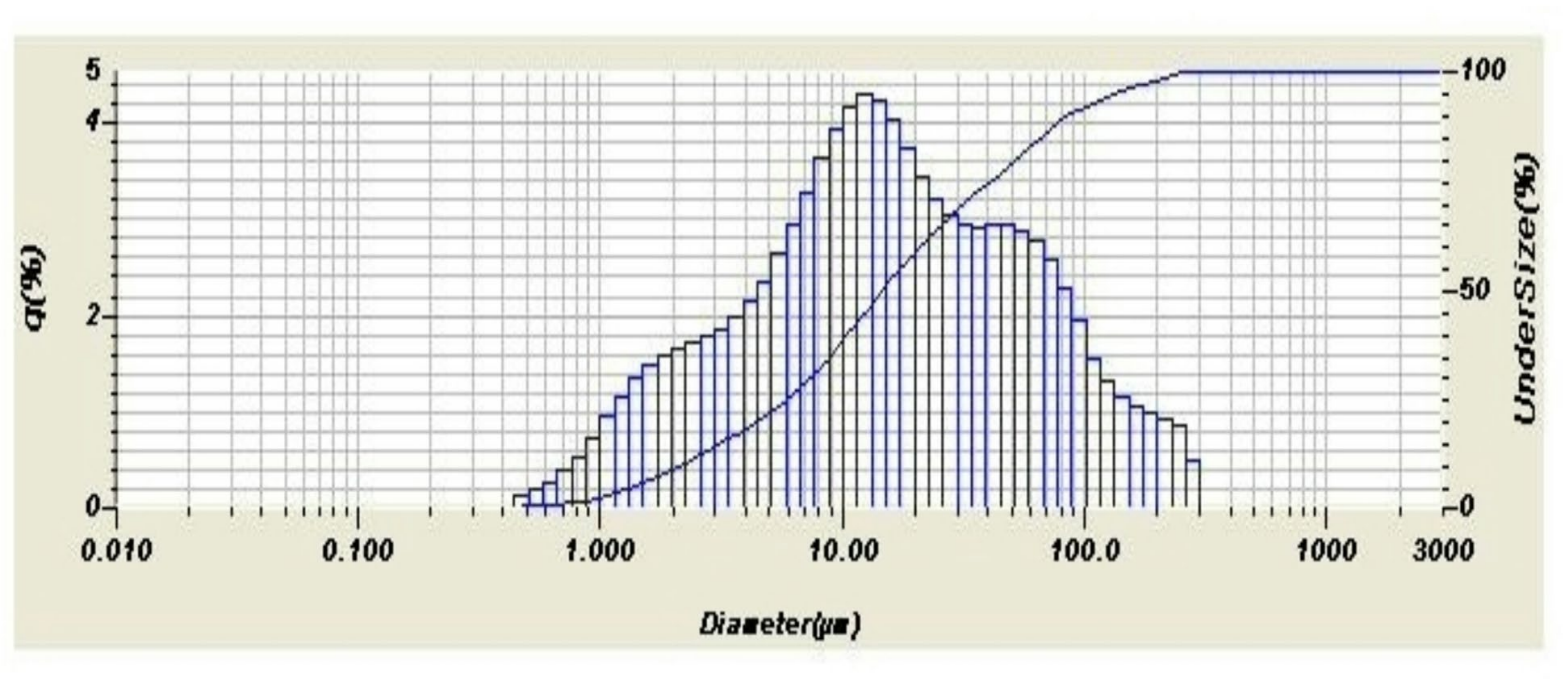
The normal curve of the particle size distribution of the ground demolition ceramic wall waste.

**Fig. 7:**
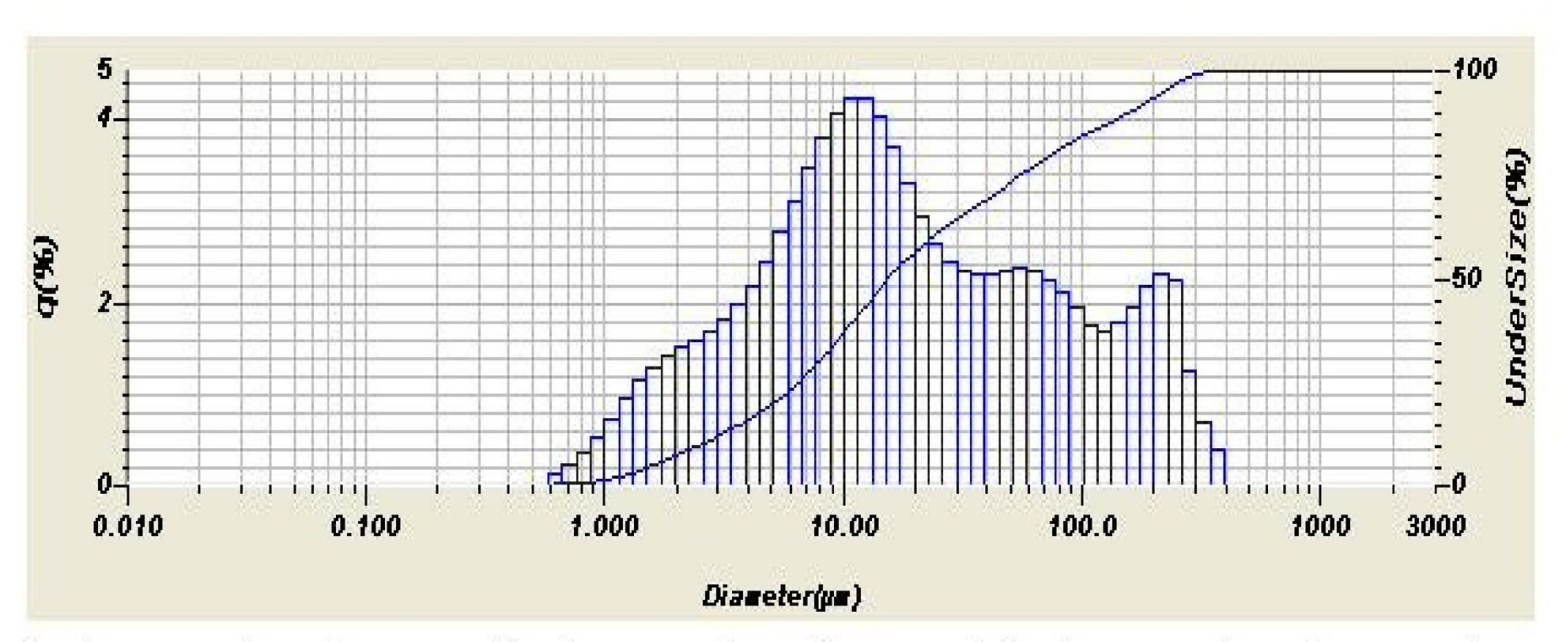
The normal curve of the particle size distribution of the ground demolition ceramic wall waste.

The laboratory grinding was ordered to be around 75 micron as particle size for both samples Figs. 6 and 7 show the particle size distribution of both samples as resulting from the analyzer. The curve in Fig. 6 shows irregular normal distribution with almost two looking like peaks. Both curves show irregular normal distribution. The particle size distribution analysis is explained by the direct and calculated parameters from the tables produced by the analyzer as shown in Table 4.

**Table 4:** Particle Size parameters obtained from Figs. 6 and 7.

Parameter	Ceramic wall demolition waste	Ground demolition ceramic waste
Mean size	34.150 μm	49.058 μm
Median size	14.845 μm	15.729 μm
Mode size	12.390 μm	12.341 μm
Diameter on cumulative of 10%	2.230 μm	2.660 μm
Diameter on cumulative of 90%	89.236 μm	162.250 μm

Comparing the particle size analysis of this sample (floor) with the previous one (wall), it can be deducted that the wall sample was ground more fine. It is expected as the floor tiles are generally more thick and harder because, due to technology, floor tiles are more green compressed and fired at higher temperatures.

### XRD investigations

**Fig. 8:**
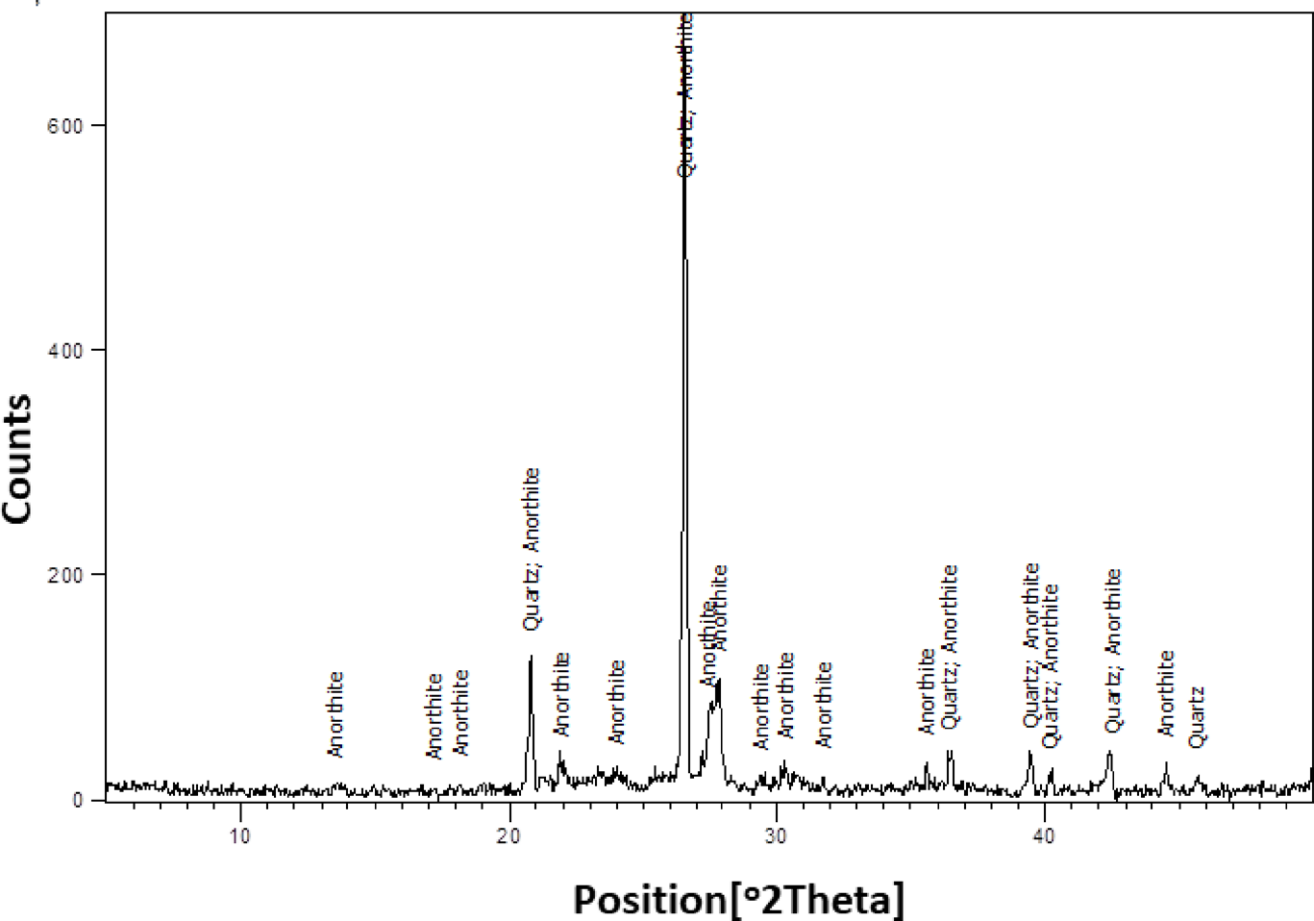
XRD of the demolition ceramic wall waste.

**Fig. 9:**
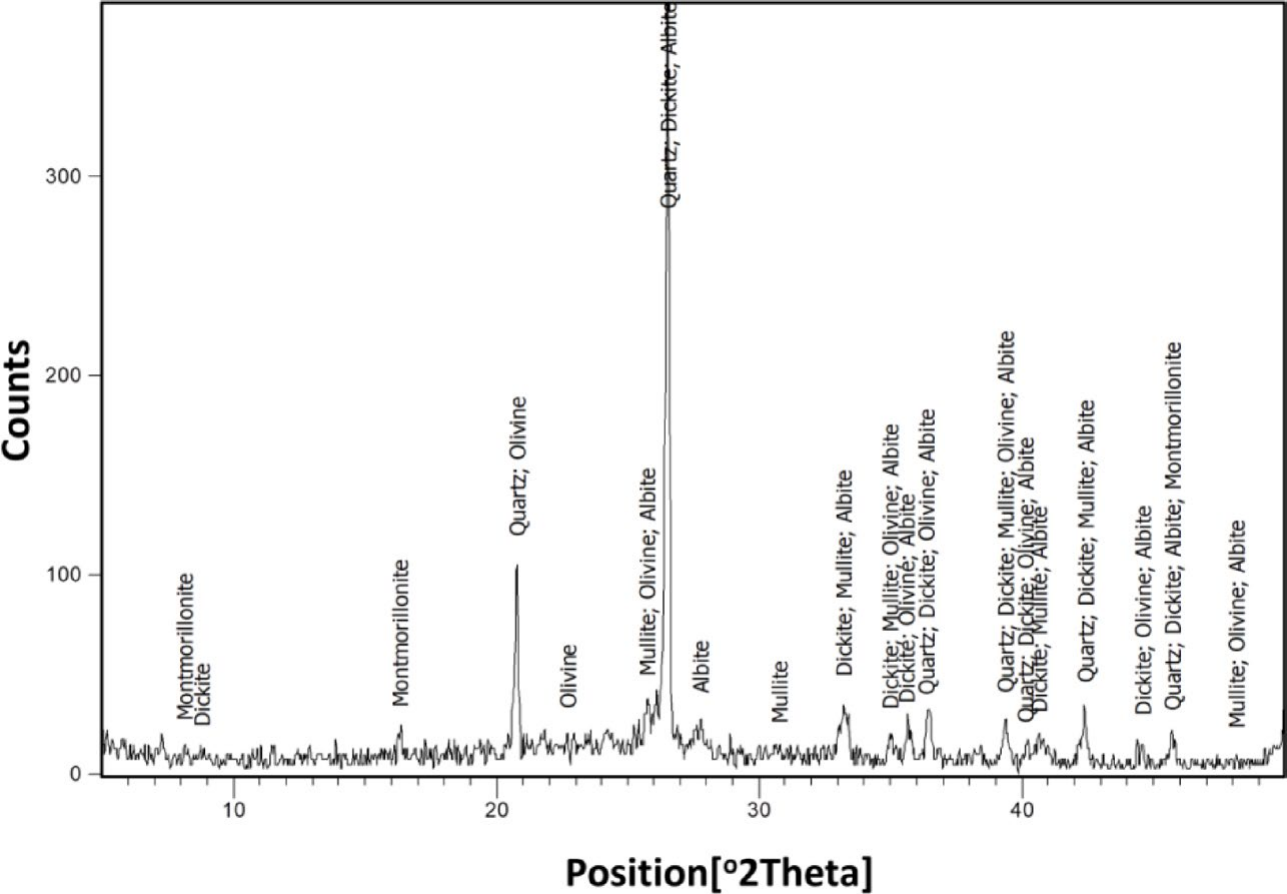
XRD of the demolition ceramic floor waste.

Mineralogical analyses of the two samples are illustrated in Figs. 8 and 9. Figure 8 reveals the presence of Quartz (SiO₂) and Anorthite ((Ca₀.₉₄Na₀.₀₆)Al₂Si₂O₈), both of which are thermally stable phases that contribute to the mechanical strength of ceramic tiles. Based on the XRF data, the low concentration of iron oxide suggests that iron is not structurally incorporated into the crystalline phases identified.

Figure 9 indicates a more diverse mineral assemblage, comprising Quartz (SiO₂), Dickite (Al₂Si₂O₅(OH)₄), Mullite (3Al₂O₃·2SiO₂), Olivine (Mg₁.₈Fe₂(SiO₄)), Albite ((Na₀.₉₈Ca₀.₀₂)(Al₁.₀₂Si₂.₉₈O₈)), and Montmorillonite ((Al(OH)₂)₀.₃₃Al₂(Si₃.₆₇Al₀.₃₃O₁₀)(OH)₂).

According to reference^34^, Olivine represents a group of silicate minerals commonly found in igneous rocks such as basalt. These minerals typically span a compositional range between forsterite (Mg₂SiO₄) and fayalite (Fe₂SiO₄), forming a solid solution series. Due to its high melting point, Olivine is recognized as a valuable refractory material^35^. The melting points of forsterite and fayalite are approximately 1900 °C and 1200 °C, respectively, and their relative proportions are often expressed in molar percentages^36^. The reddish hue observed in powder sample W2, as shown in Fig. 1, is indicative of a dominant fayalite phase. This interpretation is supported by the chemical composition in Fig. 7, which shows a higher concentration of fayalite relative to forsterite, thereby explaining the sample’s coloration. Moreover, the high proportion of quartz and feldspathic phases (anorthite/albite) in ceramic wastes, as shown in the XRD patterns (Figs. 8 and 9), suggests that these materials have excellent thermal stability and structural rigidity^37^. Mullite, which is thought to increase mechanical strength and heat resistance, appears in the waste from floor tiles as a result of high temperature firing. Partial solid solution formation, as suggested by the presence of olivine and aluminosilicate phases, is in agreement with Fe-Mg substitution that occurs during sintering. Reports indicate that burned ceramic wastes, which often contain such phase assemblages, have much improved refractory performance and durability^38,39^.

### Chemical composition: (XRF)

Table 5displays the X-ray fluorescence (XRF) study of waste derived from wall and floor tiles. The waste from wall tiles mostly consists of oxides often linked to the clay materials used in ceramic wall tile manufacturing, including silica (SiO_2_) and alumina (Al₂O₃), in addition to iron oxides, the concentration of which fluctuates according on the source of the raw materials^35–39^. The occurrence of potassium oxide (K_2_O) and sodium oxide (Na_2_O) is ascribed to minor salts generated from feldspar. Supplementary oxides including titanium (Ti), zirconium (Zr), phosphorous (P), zinc (Zn), and chromium (Cr) derive from the glaze coatings. The loss on ignition (LOI) is negligible, aligning with the fired and aged characteristics of the ceramic tiles.

Conversely, the waste from ground floor tiles also contains silica and alumina as primary components, with a negligible level of calcium oxide (CaO). Iron oxides are identified at elevated amounts, perhaps contributing to the crimson hue of the crushed powder^35^. The presence of K₂O, Na₂O, and magnesium oxide (MgO) is once more associated with salts generated from feldspar. Oxides associated with glaze, including as Ti, Zr, P, and Zn, are present, along with trace quantities of insignificant elements^40^. The LOI is low, indicating the thermally treated and aged state of the ceramic material.

**Table 5:** XRF data for Wall tiles ground waste and Floor tiles ground waste.

Sample	Waste ceramic (Wall)	Waste ceramic (Floor)
SiO_2_	64.78	60.66
Al_2_O_3_	17.80	19.50
CaO	6.63	2.31
K_2_O	3.17	2.50
Na_2_O	1.64	1.39
TiO_2_	1.30	1.46
Fe_2_O	1.29	9.11
P_2_O_5_	0.46	0.55
ZrO_2_	0.41	0.27
MgO	0.36	0.39
SO_3_	0.32	0.21
ZnO	0.25	0.11
Cr2O	0.03	0.04
SrO	0.02	0.04
Cl-	0.09	0.10
LOI	1.43	1.03
Total	99.98	99.84

### Density

**Fig. 10:**
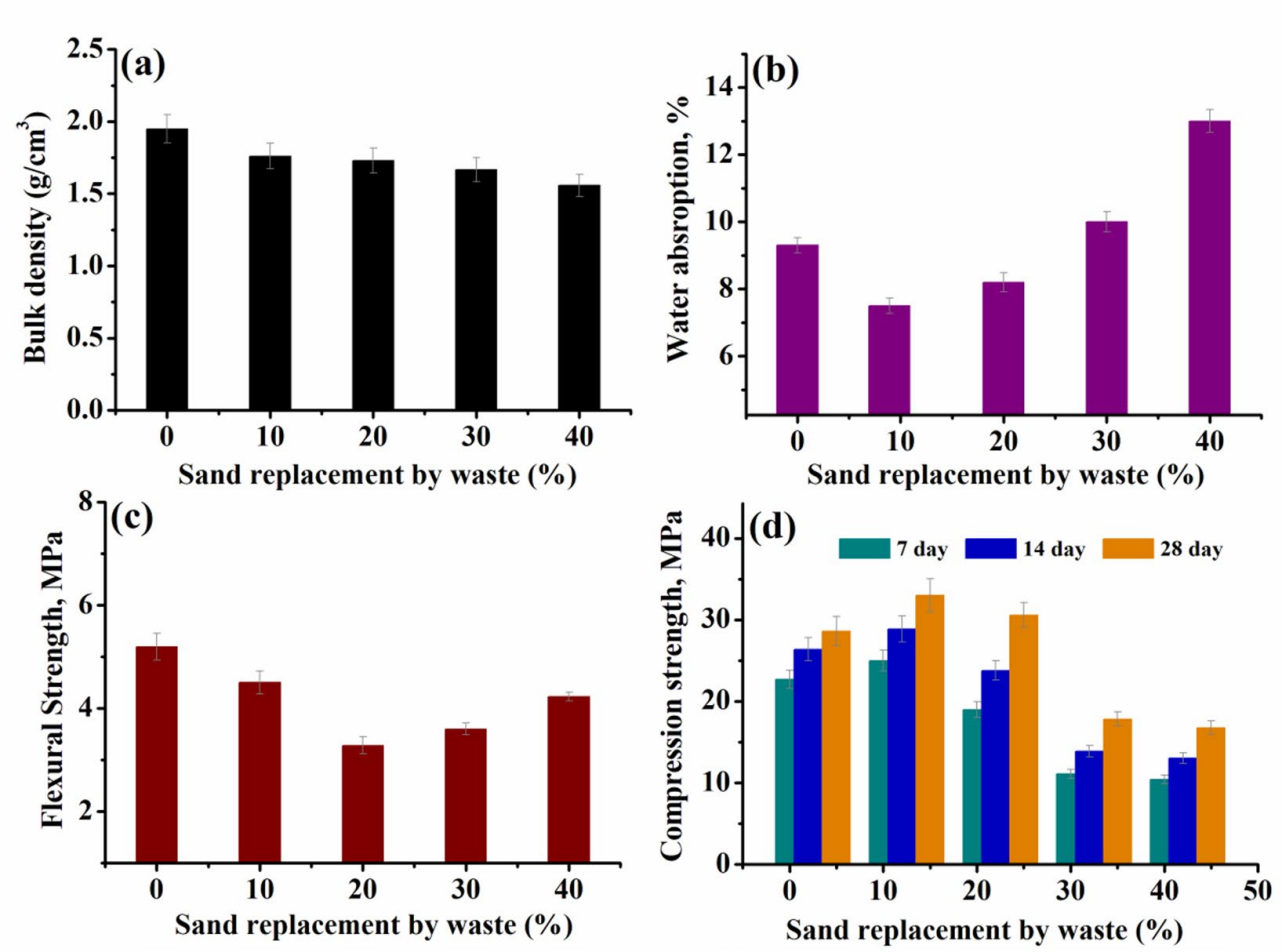
Density, water absorption, compression strength and flexural strength of concrete samples (group (I)) with waste rubber (WR) and waste ceramics (W1, W2) sand substitution.

**Fig. 11:**
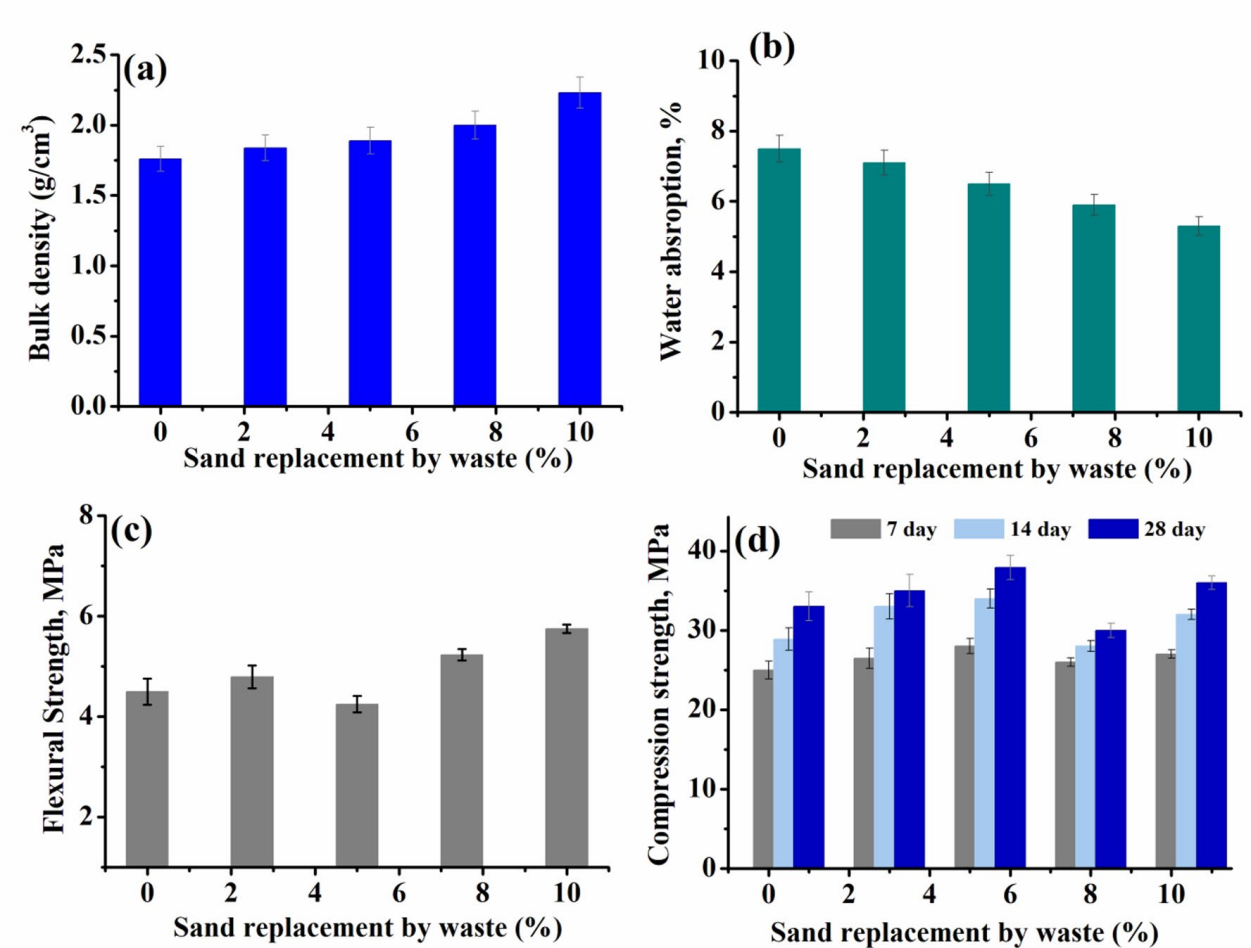
Density, water absorption, compression strength and flexural strength of concrete samples (group (II)) with constant loading of waste rubber (WR), waste ceramics (W1,W2) and varied Waste iron powder (WIP) loading.

**Fig. 12:**
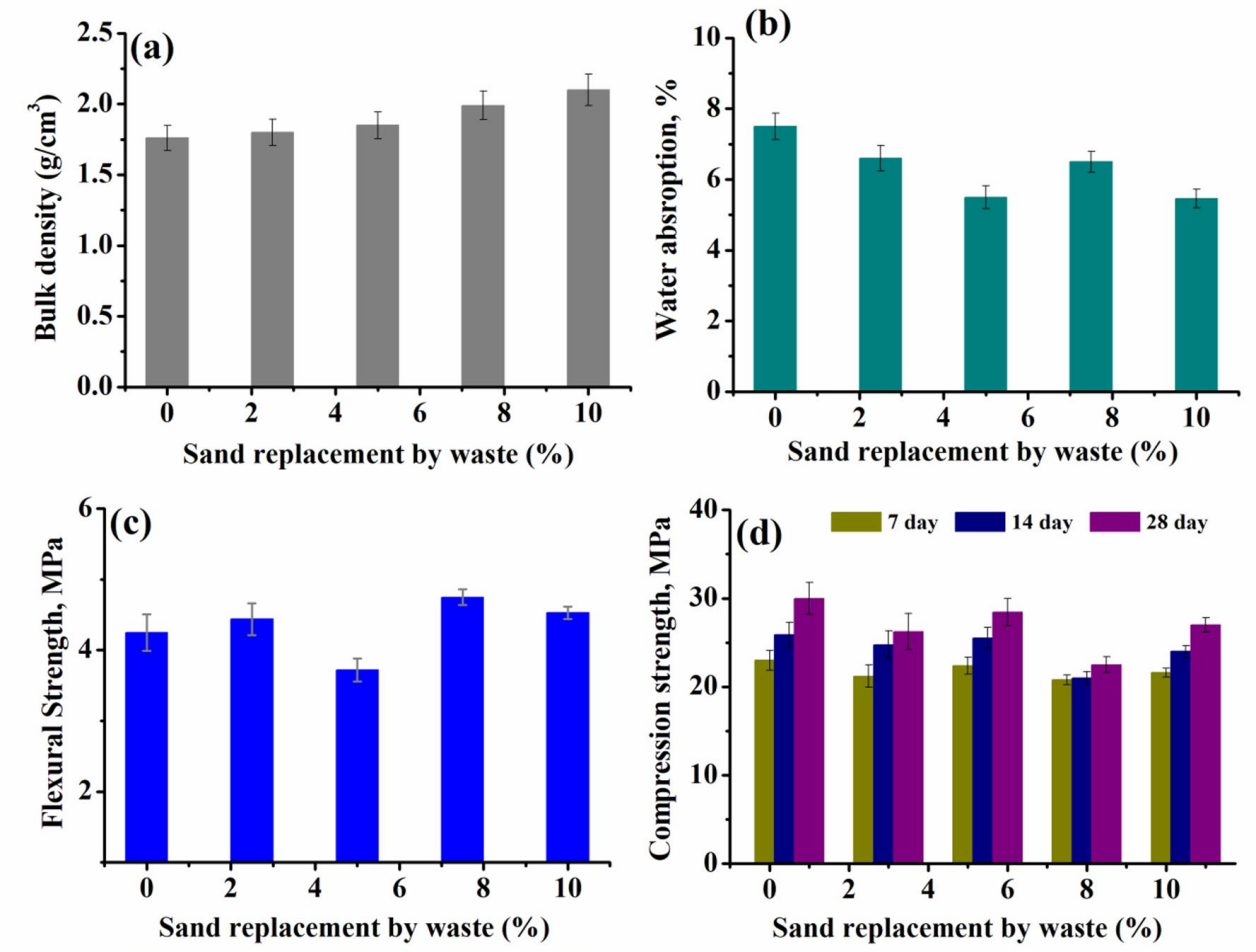
Density, water absorption, compression strength and flexural strength of concrete samples (group (III)) with constant loading of waste rubber (WR), waste ceramics (W1,W2) and varied Waste iron powder (WIP) loading and impeded metal net.

The data of density, water absorption, compression strength and flexural strength of all groups were measured and are listed in Table 6 and presented in Figs. 10, 11 and 12a. The density of the reinforcing cement mortar sample was determined after a cure period of 28 days. For the first group (Group I), in Fig. 10a.The density of all samples, record a decrement upon increasing the loading of waste rubber and both ceramic wastes, compared to the reference cement mortar^40,41^. This decrease may be due to the presence of.

**Table 6:** Density of (g/cm^3^) different concrete samples in groups I, II and III.

Group No. I		Group No. II		Group No. III	
Waste (%)	Density (g/cm^3^)	Waste (%)	Density (g/cm^3^)	Waste (%)	Density (g/cm^3^)
0	1.95	0	1.76	0	1.76
10	1.76	2.5	1.838	2.5	1.8
20	1.73	5	1.89	5	1.85
30	1.667	7.5	2	7.5	1.99
40	1.557	10	2.23	10	2.1

WR, which results in the production of lightweight samples. However, it increased as the percentage of waste iron powder (WIP) increased (Fig. 11a) compared to the reference sample containing 10% of WR, W1 and W2. This increase is a result of the increased density of iron powder compared to other materials used. On the other hand the density, doesn’t demonstrate a significant change after impeding the metal net (Fig. 12a).

### Water absorption

The water absorption of concrete samples was determined, listed in Table 7 , 8 and illustrated in Figs. 10, 11 and 12 b. Clearly, for samples in Fig. 10b-group (I), an increase in waste level causes a corresponding increase in water absorption owing to increased porosity^42^. The increase of waste content from 10% to 40% increased the water absorption by about 39%. On the other hand in Fig. 11b- group (II), the water absorption decreased with increasing waste iron powder (WIP) percentage as compared to the blank sample^43^. The values of water absorption for Group (III) in Fig. 12b- are comparable to those for Group (II). However, the presence of WIP without/with metal net seems likely to improve concrete mixture microstructure and water absorption as well^42^.

**Table 7:** Water absorption (%) different concrete samples in groups I, II and III.

Group No. I		Group No. II		Group No. III	
Waste (%)	Water absorption,%	Waste (%)	Water absorption,%	Waste (%)	Water absorption,%
0	9.3	0	7.5	0	7.5
10	7.5	2.5	7.1	2.5	6.6
20	8.2	5	6.5	5	5.5
30	10	7.5	5.9	7.5	6.5
40	13	10	5.3	10	5.46

### Flexural strength

Figures 10, 11 and 12c, show how the substitution of sand by waste affects the flexural strength of the concrete samples.The measured data of the flexural strength are also listed in Table 7. For group (I), the measured values of flexural strength decreased at the initial loading up to 20%, followed by significant increase at higher waste loading. Further, addition of WIP (Group II) and WIP/metal net (Group III) to cement mortar enhance the flexural strength values compared to the blank sample^44^. This increase was caused by the WIP particles’ strong bonds with the cement matrix. At high percentage of iron powder in addition to the presence of which is known to be more flexible than cement hydrate and to have good elasticity and adhesion, the flexural strength of specimens at 10% WIP with WR, W1 and W2 was higher than the other counterparts, even when compared to reference cement mortar^45^ (Table 8).

**Table 8:** Water absorption (%) different concrete samples in groups I, II and III.

Group No. I		Group No. II		Group No. III	
Waste (%)	Flexural strength (MPa)	Waste (%)	Flexural strength (MPa)	Waste (%)	Flexural strength (MPa)
0	5.20	0	4.5	0	4.25
10	4.50	2.5	4.79	2.5	4.44
20	3.28	5	4.25	5	3.72
30	3.62	7.5	5.23	7.5	4.75
40	4.23	10	5.75	10	4.53

### Compressive strength

Compressive strength of the reinforcing cement mortar samples was assessed at 7, 14 and 28 days after curing, as depicted in Figs. 10, 11 and 12d. For all samples, it is found that, as the curing time increased (7–28 day), there were variations in the compressive strength outcomes^44^. The compressive strength of Group (I) -Fig. 10d, increased up to 20% then decreased. Once the replacement exceeds 20%, the results indicate that there will be a steady reduction in strength due to ongoing partial replacement. This observed pattern can be explained by the decrease in the bonding strength between the surface of WR, W1 and W2 aggregate and the cement mortar. This decrease is also caused by the disparity in particle size, shape, and surface roughness between the sand and other wastes. However, WIP has good impact on the compressive strength of Group (II) and Group (III) Figs. 11 and 12d. This enhancement in strength can be attributed to the increased density resulting from the addition of iron powder waste and improvement concrete mixture microstructure^44,45^.

**Fig. 13:**
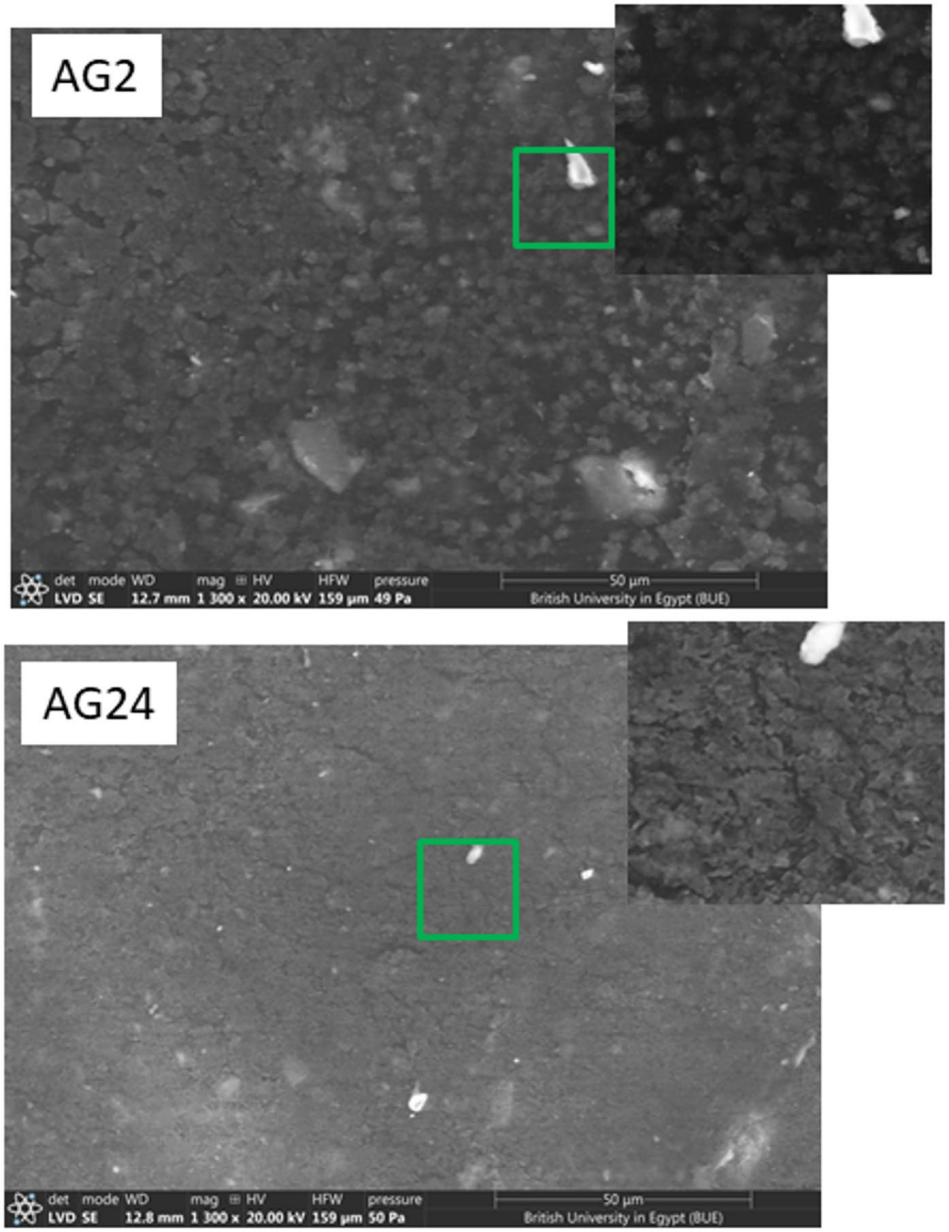
SEM micrographs of sample AG5 and AG24.

However, one can reach the conclusion that, the incorporation of ceramic waste, rubber particles, and waste iron powder induces notable modifications in the composite microstructure, which are directly associated with variations in density, water absorption, and mechanical performance. When natural sand is substituted with rubber and ceramic waste, the resulting particles-characterized by irregular morphology, reduced stiffness, and weaker interfacial bonding with cement hydrates-promote increased porosity and diminished packing efficiency. This microstructural discontinuity accounts for the reduction in density and the concomitant rise in water absorption observed in Group I specimens at higher waste contents^46,47^. Conversely, the addition of waste iron powder significantly alters the cementitious matrix configuration. The nanoscale to submicron-sized iron particles act as effective micro-fillers, occupying capillary pores and interstitial spaces between cement hydrates and ceramic inclusions. This filler effect facilitates a transition from the porous, heterogeneous structure of Group I to the denser, more homogeneous matrix observed in Group II, as confirmed by SEM analysis (Fig. 13), which will be elaborated upon in a subsequent section. The densification of the matrix enhances stress transfer across the interfacial transition zones, thereby improving both compressive and flexural strengths^46–48^.

### Microstructural evaluation

Scanning Electron Microscopy (SEM) was employed to assess the surface morphology and microstructural characteristics of representative samples AG2 (Group I) and AG24 (Group II**).** The micrographs of the two samples are depicted in Fig. 13. Sample AG2 exhibited a relatively coarse and porous microstructure. The SEM image revealed irregular particle distribution and visible voids, indicating suboptimal packing density. In contrast, sample AG24, displayed a significantly more compact and homogeneous microstructure. The SEM image showed smoother surfaces with fewer voids and enhanced particle dispersion. The presence of WIP facilitated better packing and stronger interfacial adhesion, forming a cohesive cementations’ network. This densification is expected to improve mechanical properties and reduce water absorption as discussed previously. The fine iron particles likely acted as micro-fillers, bridging gaps between larger aggregates and enhancing the continuity of the matrix, consistent with findings from Huseien et al^49^. The improved microstructure of AG24 correlates with enhanced mechanical performance. Flexural and compressive strength tests confirmed that AG24 outperformed AG5, despite both having equivalent total waste content. This suggests that the inclusion of WIP not only compensates for the reduction in sand but also contributes to matrix refinement and strength development, as supported by similar studies on ceramic waste-based composites^49,50^.

### **Electrical conductivity measurements**

**Fig. 14:**
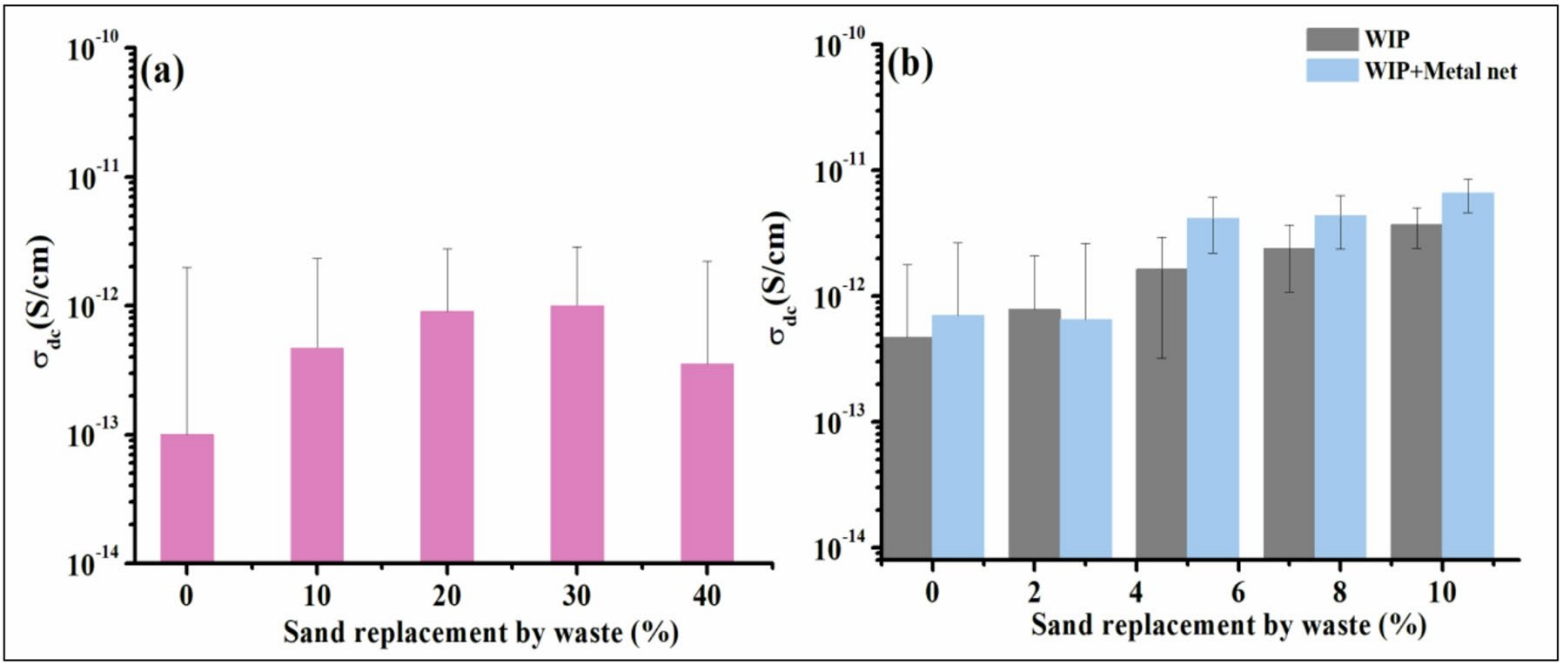
The electrical conductivity of all cements concrete samples.

The electrical conductivity of all cement concrete samples is measured and the data are illustrated in Fig. 14. From Fig. 11a of Group (I) samples, the conductivity increased up to 30% loading then decreased. Figure 11b for samples in for group (I and II) after the addition of WIP different loading and presence of metal net proves to have positive impact on the detected values. The recorded values of the samples lies in the range of 10^− 13^ − 10^− 11^ S/cm which is the demanded range of antistatic applications that finding recommend such samples to be used as antistatic floor^51,52^.This result find further justification through the EMI shielding studies presented in the next section.

### EMI Shielding effectiveness

Wire mesh shielding exploits the electrical conductivity of metallic meshes to attenuate electromagnetic waves within enclosed regions, thereby ensuring stable operation of electronic, communication, medical, and aerospace equipment. The EMI shielding effectiveness (SE) of cementitious composites arises from a synergistic combination of reflection, absorption, and multiple internal scattering mechanisms^53,54^. Reflection is primarily governed by conductive phases that induce impedance mismatch at the air–material interface. In the present study, the incorporation of waste iron powder and wire mesh markedly increases the electrical conductivity of the cement matrix, thereby enhancing the reflection component of EMI shielding^54^.

**Fig. 15:**
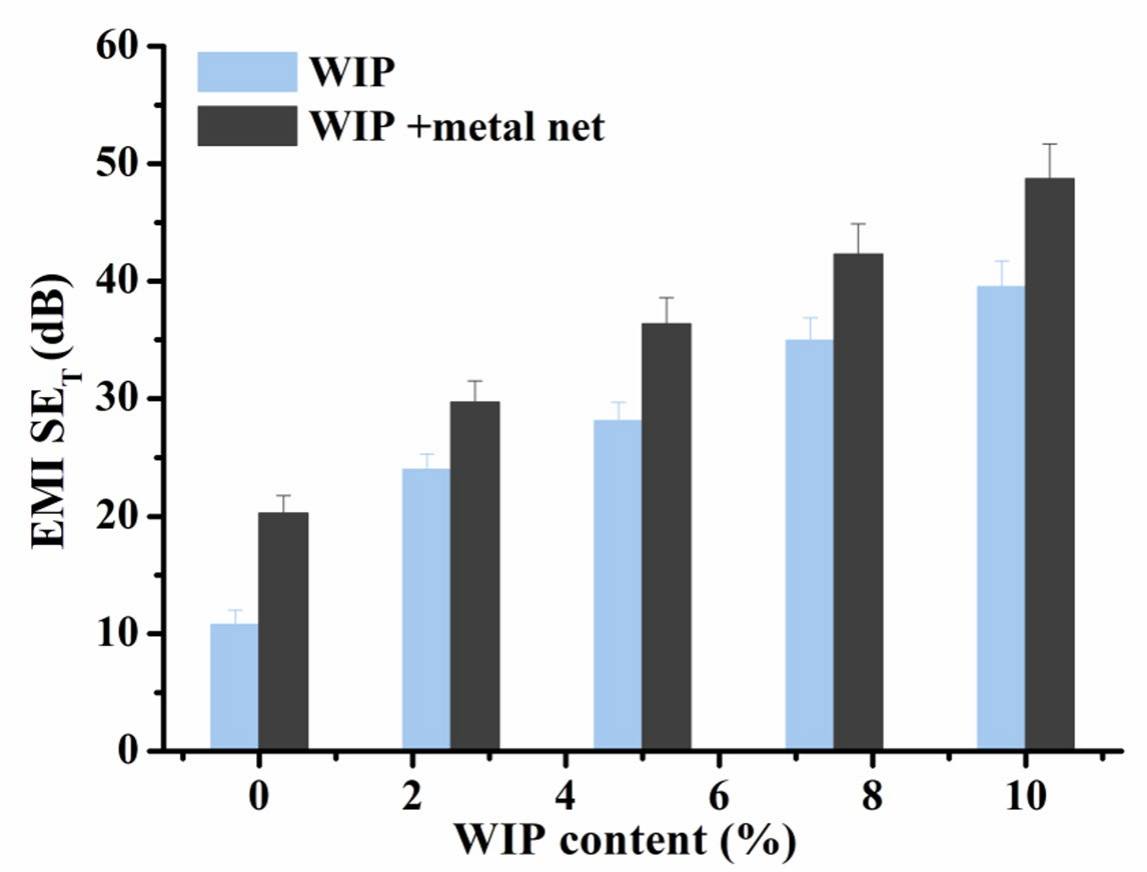
Total EMI shielding effectiveness for concrete samples with different ratios of WIP and WIP/metal net.

Figure 15 displays the total EMI SE_T_ shielding effectiveness of Group (II) and Group (III) at fixed frequency (10 GHz) for comparison. Figure 15 demonstrates that the cement concrete with 10% waste (WR, W1 and W2) has an almost negligible SE ~ 10.8 dB, arises from the waste inclusions. This value of blank sample rose to ~ 20 dB once the metal net is embedded within the cement matrix. Since, the values of the blank sample with metal net above 20 db. This indicates that it is capable of efficiently blocking 99% of electromagnetic waves, making it suitable for industrial and commercial applications requiring EMI shielding materials. However, it is noticed that, the inclusion of WIP particles can significantly enhance the EMI shielding capabilities of the insulating concrete. The SE_T_ values climb to 23 when 2.5% WIP is included, and then rise to 39.31 when 10% WIP is incorporated. Moreover, The SE_T_ values enhanced for Group (III) samples with metal net. It reaches ~ 48 dB at 10% WIP with metal net.

In the cementitious matrix, the conductive iron particles create interconnected or semi-interconnected networks that allow electromagnetic energy to be dissipated through Ohmic losses. Iron’s magnetic properties also contribute to magnetic loss mechanisms, which increase the material’s energy attenuation. This is demonstrated by the shielding efficiency gradually increasing as WIP content increases, which are in good agreement with the observed increase in electrical conductivity. The heterogeneous and porous architecture of the composites increases the significance of multiple internal scattering. Iron powder, rubber inclusions, and ceramic particles all produce a variety of surfaces with different dielectric characteristics. At these interfaces, incident electromagnetic waves repeatedly reflect and refract, lengthening their propagation route inside the material and encouraging more energy dissipation. Samples with WIP have a refined microstructure with evenly dispersed conductive particles, which promotes repetitive internal scattering, according to SEM measurements. The overall shielding efficiency is greatly increased by the addition of a wire mesh, which creates a macroscopic conductive network that serves as a major reflective barrier. The distributed iron particles and the wire mesh work together to provide a hybrid shielding mechanism in which multiple scattering and absorption predominate in the bulk while reflection predominates at the surface. As a result, samples with both wire mesh and WIP attained shielding efficacy values of up to ~ 48 dB, which is equivalent to more than 99.99% attenuation of incident electromagnetic radiation.

It can be concluded that these concrete can effectively blocking 99–99,999% of electromagnetic waves. That is, they can be efficiently used as cement tile for the roof of building with cell phone towers. It can act as a shield to protect against the effects of EMI radiation for residents of the buildings. Finally, from the above results, it was found that the produced cement tile complies with the Egyptian standard : 269-2/2021, the cement tiles, part 2: cement tiles for external use^55^, which harmonize and is referenced to the European standard EN 13748-2/2004^56^.

### Quantitative sustainability assessment

Because virgin aggregate extraction and processing are avoided, there is a discernible decrease in embodied CO_2_ emissions when natural sand is partially substituted with ceramic demolition waste, waste rubber, and waste iron powder. Mixtures including up to 40 weight% waste show an estimated 25–35% reduction in CO_2_ emissions compared to traditional cement mortar tiles, based on stated emission factors for natural sand (≈ 4–6 kg CO_2_ t⁻¹) and using a simplified life-cycle approach^57,58^.

From an economic standpoint, using locally accessible waste materials reduces aggregate-related material costs by an estimated 20–30% because the acquisition prices of iron by-products and ceramic and rubber wastes are lower than those of natural sand. Additionally, the improved EMI shielding performance offers indirect cost advantages by lowering the need for additional metallic shielding systems.

Using a strength-to-density index, a density-strength trade-off analysis was carried out to assess performance efficiency. Rubber-containing mixes lower density, but strength is compromised by excessive replacement. Formulations using 10% waste iron powder, on the other hand, attain the ideal balance and show enhanced flexural and compressive strength with just a slight increase in density. The proposed composites exhibit both material efficiency and functional performance, as evidenced by their better strength-to-density ratio as compared to the reference mortar.

## Conclusion

Ceramic waste materials sourced from the renovation of bathrooms and kitchens in an old building were successfully granulated, and their particle size distribution was characterized. A comparative analysis between floor and wall tile waste revealed that the wall-derived sample exhibited finer particle sizes.

This study successfully shows that recovered tyre rubber, waste iron powder and ceramic demolition waste may be used to produce sustainable, multipurpose outdoor cement tiles. Ceramic floor and wall tile wastes were ground and characterized to validate their acceptability as fine aggregate alternatives; wall tile waste showed finer particle sizes because of variations in fire and manufacturing circumstances. Because rubber particles are lightweight and have more porosity, adding rubber and ceramic debris resulted in a decrease in density and an increase in water absorption. However, by increasing matrix densification and strengthening interfacial interaction within the cementitious system, the addition of waste iron powder considerably reduced these effects. Compressive and flexural strengths were thereby significantly increased, with 10 weight% WIP showing the best results. WIP functioned as an efficient micro-filler, decreasing voids and encouraging a more uniform and compact cement matrix, according to microstructural research. When WIP was added, the composites’ electrical characteristics changed from insulating to antistatic, reaching conductivity levels appropriate for functional flooring applications. Additionally, a significant improvement in EMI shielding effectiveness-up to ~ 48 dB in the X-band frequency range-was made possible by the synergistic action of waste iron powder and wire mesh reinforcement, allowing for effective electromagnetic radiation attenuation. Electrical conductivity measurements of all cementitious samples fell within the range of 10^− 13^ to 10^− 11^ S/cm, aligning with the requirements for antistatic applications. These findings support the suitability of such composites for use as antistatic roof-shielding tiles. Electromagnetic interference (EMI) shielding studies revealed that the incorporation of WIP significantly improved the shielding effectiveness of the insulating concrete. The most pronounced enhancement was observed in samples reinforced with metal mesh, which achieved EMI attenuation levels of 99% to 99.999%. This confirms the potential of WIP–metal mesh composites as effective EMI shielding materials. This work provides a practical and environmentally responsible approach to converting construction, industrial, and rubber wastes into high-performance building materials with added functional value, contributing to circular economy and sustainable infrastructure development. Moreover, developed tiles comply with Egyptian and European standards for external cement tiles, demonstrating their suitability for sustainable construction applications, particularly for roofing and flooring in environments exposed to electromagnetic pollution. This work highlights an effective pathway for converting multiple waste streams into high-value, multifunctional building materials.

## Data Availability

The datasets used and/or analyzed during the current study available from the corresponding author on reasonable request.

## References

[1] Ministry of Environment, Egypt. National Strategy for C&D Waste Management. WMRA. (2020).

[2] Kondarage, Y. G., Pitawala, H. M. J. C., Kirushanthi, T., Edirisinghe, D. & Etampawala, T. N. Ceramic waste-based natural rubber composites: An exciting way for improving mechanical properties. *J. Adv. Chem. Sci.* **4**, 576–582 (2018).

[3] Tokareva, A., Kaassamani, S. & Waldmann, D. Using ceramic demolition wastes for CO₂ reduced cement production. *Constr. Build. Mater.* **426**, 135980 (2024).

[4] de Matos, P. R. et al. Utilization of ceramic tile demolition waste as supplementary cementitious material: An early age investigation. *J. Build. Eng.* **38**, 102187 (2021).

[5] Sadek, D. M., Amin, S. K. & Youssef, N. F. Blended cement utilizing ceramic wall tiles waste. In Ekolu, S. O. et al. (eds.) *Construction Materials and Structures*. 152–161 (IOS, 2014).

[6] Huseien, G. F. et al. Geopolymer mortars as sustainable repair material: A comprehensive review. *Renew. Sustain. Energy Rev.* **80**, 54–74 (2017).

[7] Keshavarz, Z. & Mostofinejad, D. Porcelain and red ceramic wastes used as replacements for coarse aggregate in concrete. *Constr. Build. Mater.* **195**, 218–230 (2019).

[8] Huseien, G. F. et al. Sustainability of recycling waste ceramic tiles in the green concrete industry: A comprehensive review. *Buildings* **15**, 2406 (2025).

[9] Korat, A., Amin, M. & Tahwia, A. M. A comprehensive assessment of ceramic wastes in ultra-high-performance concrete. *Innov. Infrastruct. Solut.* **10**, 28 (2025).

[10] de Matos, P. R. et al. Utilization of ceramic tile demolition waste as supplementary cementitious material: An early age investigation. *J. Build. Eng.* **38**, 102187 (2021).

[11] Mandal, A. & Rajput, S. P. S. Advances in alkali-activation of ceramic waste-based pozzolana in concrete and mortar: A comprehensive review. *Waste Biomass Valoriz.* **16**, 3309–3330 (2025).

[12] Saiz Martínez, P., Ferrández, D., Melane-Lavado, A. & Zaragoza-Benzal, A. Characterization of three types of recycled aggregates from different construction and demolition waste: An experimental study for waste management. *Int. J. Environ. Res. Public Health*. **20**, 3709 (2023). 36834403 10.3390/ijerph20043709 PMC9963922

[13] Xiao, J. et al. Recycled aggregate concrete design, application and challenges. *Nat. Rev. Clean Technol.*10.1038/s44359-025-00125-2 (2026).

[14] Shafik, E. S., Tharwat, C. & Abd El Messieh, S. L. Utilization study on red brick waste as novel reinforcing and economical filler for acrylonitrile butadiene rubber composite. *Clean Technol. Environ. Policy* **25**, 1605–1615 (2023).

[15] Khan, R., Iqbal, S., Soliyeva, M., Alid, A. & Elboughdiri, N. Advanced clay-based geopolymer: Influence of structural and material parameters on its performance and applications. *RSC Adv.* **15**, 12443. 10.1039/d4ra07601j (2025). 40264885 10.1039/d4ra07601j PMC12012607

[16] Heath, A. et al. Contested terrain: Explaining divergent patterns of public opinion towards immigration within Europe. *J. Ethn. Migr. Stud.* **46**, 475–488 (2020).

[17] Buddhacosa, N. et al. Crush behaviour and vibration damping properties of syntactic foam incorporating waste tyre-derived crumb rubber. *J. Mater. Res. Technol.* **26**, 3214–3233 (2023).

[18] Al-Subari, L., Ekinci, A. & Aydın, E. The utilization of waste rubber tire powder to improve the mechanical properties of cement-clay composites. *Constr. Build. Mater.* **300**, 124306. 10.1016/j.conbuildmat.2021.124306 (2021).

[19] Siddika, A. et al. Properties and utilizations of waste tire rubber in concrete: A review. *Constr. Build. Mater.* **224**, 711–731 (2019).

[20] Wang, Q. et al. Site selection optimization of reverse logistics network for waste tires. Wireless Communications and Mobile Computing. e5438290. (2022). (2022).

[21] Gu, W. et al. Environmentally friendly and multifunctional shaddock peel-based carbon aerogel for thermal insulation and microwave absorption. *Nano-Micro Lett.* **13**, 102 (2021). 10.1007/s40820-021-00635-1 PMC8021664 34138342

[22] Liu, X., Sun, D., Wang, L. & Guo, B. Sodium humate functionalized graphene and its unique reinforcement effects for rubber. *Ind. Eng. Chem. Res.* **52**, 14592–14600 (2013).

[23] Lin, Y. et al. A highly stretchable and sensitive strain sensor based on graphene-elastomer composites with a novel double-interconnected network. *Journal of Materials ChemistryC* **4**, 6345–6352 (2016).

[24] Rafiee, M. et al. Enhanced mechanical properties of nanocomposites at low graphene content. *ACS Nano* **3**, 3884–3890 (2009). 19957928 10.1021/nn9010472

[25] Zhao, B. et al. Achieving wideband microwave absorption properties in PVDF nanocomposite foams with an ultra-low MWCNT content by introducing a microcellular structure. *Journal of Materials ChemistryC* **8**, 58–67 (2020).

[26] Weldemhret, T. G., Park, Y. T. & Song, J. I. Recent progress in surface engineering methods and advanced applications of flexible polymeric foams. *Adv. Colloid Interface Sci.*10.1016/j.cis.2024.103132 (2024). 38537566 10.1016/j.cis.2024.103132

[27] Mu, Z., Xie, P. & Alshammari, A. From structure to function: Innovative applications of biomass carbon materials in microwave absorption. *Adv. Compos. Hybrid. Mater.* **7**, 220 (2024).

[28] Khade, V. & Wuppulluri, M. Microwave absorption performance of flexible porous PVDF–MWCNT foam in the X-band frequency range. *ACS Omega*. **9**, 35364–35373 (2024). 39184473 10.1021/acsomega.4c00995 PMC11339829

[29] Lan, D., Zhou, H. & Wu, H. A polymer sponge with dual absorption of mechanical and electromagnetic energy. *J. Colloid Interface Sci.* **633**, 92–101 (2023). 36436351 10.1016/j.jcis.2022.11.102

[30] Li, Y. et al. Multifunctional organic-inorganic hybrid aerogel for self-cleaning, heat-insulating, and highly efficient microwave absorbing material. *Adv. Funct. Mater.* **29**, 1807624 (2019).

[31] Zachariah, S. M., Antony, T., Grohens, Y. & Thomas, S. From waste to wealth: A critical review on advanced materials for EMI shielding. *J. Appl. Polym. Sci.* **139**, e52974 (2022).

[32] Shafik, E. S. et al. Conductive EPDM hybrid composites for electromagnetic interference shielding (EMI). *KGK-Kautschuk Gummi Kunststoffe*. **77**, 47–55 (2024).

[33] ASTM C642–1997. Standard test method for density, absorption, and voids in hardened concrete. *ASTM International* (1997).

[34] Zachariah, S. M., Antony, T., Grohens, Y. & Thomas, S. From waste to wealth: A critical review on advanced materials for EMI shielding. *J. Appl. Polym. Sci.* **139**, e52974 (2022).

[35] Geology.com & Britannica.com. Minerals: Olivine. Available: www.britannica.com Accessed (2023).

[36] Wikipedia & Olivine Fayalite. [Online]. Available: en.m.wikipedia.orgAccessed (2023).

[37] Sánchez-Soto, P. J., Eliche-Quesada, D., Martínez-Martínez, S., Pérez-Villarejo, L. & Garzón, E. Study of a waste kaolin as raw material for mullite ceramics and mullite refractories by reaction sintering. *Materials* **15**, 583 (2022). 35057297 10.3390/ma15020583 PMC8780162

[38] El-Maghraby, M. S., Abd El Ghaffar, N. I. & Ismail, A. I. M. The influence of alkaline granite addition on ceramic body sintering. *Egypt. J. Chem.* **66**, 65–76 (2023).

[39] Ferrara, L., Deegan, P., Pattarini, A., Sonebi, M. & Taylor, S. Recycling ceramic waste powder: Effects of its grain size distribution on fresh and hardened properties of cement pastes/mortars formulated from SCC mixes. *J. Sustainable Cement-Based Mater.* **8**, 1–16 (2019).

[40] Sheraz, M. et al. Fresh and hardened properties of waste rubber tires based concrete: A state of the art review. *SN Appl. Sci.* **5**, 119 (2023).

[41] Hill, T., Henderson, N., Sampson, D. & Sedlmair, J. Analytical X-ray techniques for chemical and structural characterization of ceramics. *Am. Ceram. Soc. Bull.* **102**, 34–42 (2023).

[42] Alotaibi, J. G., Alajmi, A. E., Alsaeed, T., Khalaf, J. A. & Yousif, B. F. On the incorporation of waste ceramic powder into concrete. *Front. Mech. Eng.* **10**, 1469727 (2024).

[43] Inbanila, T. & Priyadharshini, D. Effect of iron powder on strength of binary blended concrete.*Int. J. Recent Technol.Eng.* **8**,2277–3878 (2019).

[44] Herki, B. M. A. Strength and absorption study on eco-efficient concrete using recycled powders as mineral admixtures under various curing conditions. *Recycling* **9**, 99 (2024).

[45] Largeau, M. A., Mutuku, R. & Thuo, J. Effect of iron powder (Fe₂O₃) on strength, workability, and porosity of the binary blended concrete. *Open J. Civil Eng.* **8**, 411–425 (2018).

[46] Ikotun, J.O., Adedeji, P.O. & Babafemi, A.J.A comprehensive review on the performance of low-carbon ceramicwaste powder as cement replacement material in concrete. *Applied Sciences* **15**, 6037 (2025).

[47] Ahmad, J., Sabri, M.M., Majdi, A., Alattyih, W., Khan, I. & Alam, M.Durability and microstructure aspects ofsustainable concrete made with ceramic waste: A review.*Frontiers in Materials* **11**, 1508989 (2025).

[48] Khouadjia, M.L.K., Bensalem, S., Belebchouche, C., Boumaza, A., Hamlaoui, S. & Czarnecki, S. Sustainablegeopolymer tuff composites utilizing iron powder waste: Rheological and mechanical performance evaluation.*Sustainability* **17**, 1240 (2025).

[49] Ouda, A.S., Sanad, S.A. & Abdel-Moniem, S.M.Environmental valorization of iron-laden waste: Dual application inwastewater treatment and evaluation of the physico-mechanical and microstructural performance of cementitiouscomposites. *Environmental Science and Pollution Research* **32**, 23009–23028 (2025). 10.1007/s11356-025-36955-7 41037249

[50] Huseien, G.F., Sam, A.R.M., Mirza, J., Tahir, M.M., Asaad, M.A., Ismail, M. & Shah, K.W.Waste ceramic powderincorporated alkali activated mortars exposed to elevated temperatures: Performance evaluation.*Construction andBuilding Materials* **187**, 307–317 (2018).

[51] Kondarage, Y.G., Pitawala, H.M.J.C., Kirushanthi, T., Edirisinghe, D. & Etampawala, T.N. Ceramic waste-basednatural rubber composites: An exciting way for improving mechanical properties.*Journal of Advanced ChemicalSciences* **4**,576–582 (2018).

[52] Senthamarai, R., Manoharan, P.D. & Gobinath, D. Concrete made from ceramic industry waste: Durabilityproperties. *Construction and Building Materials* **25**,2413–2419 (2011).

[53] Ahmed M. Khalil, Mohammad L. Hassan, Azza A. Ward Novel nanofi brillated cellulose/polyvinylpyrrolidone/silvernanoparticles fi lms with electrical conductivity properties.*Carbohydrate Polym.* **157**, 503–511(2017). 10.1016/j.carbpol.2016.10.008 27987955

[54] El badry, H., khalaf, A.I., Ward, A.A. et al. Ionic liquid–modifi ed conductive polymer composites: a route to high-permittivity, Electrostatic Discharge (ESD) - safe materials. *J Polym Res* **32**, 453 (2025).

[55] Li, Y., Chen, W., Li, L., Lai, Z., He, X., Su, Y., Zheng, Z. & Strnadel, B. Simultaneously enhanced mechanical andelectromagnetic interference shielding properties of steel slag–recycled carbon fi ber cementitious composites viawet-grinding process. *Materials and Structures* **56** 182 (2023).*.*

[56] Kim, S., Jang, Y.S., Oh, T., Lee, S.K. & Yoo, D.-Y. Electromagnetic interference shielding eff ectiveness of multi-cracked strain-hardening cementitious composites (SHCC).*Archives of Civil and Mechanical Engineering* **23** 179(2023).*.*

[57] Egyptian Standard ES 269-2/2021:*Cement Tiles – Part 2: Cement Tiles for External Use. Egyptian Organizationfor Standardization and Quality (EOS), Cairo*, (2021).

[58] European Standard EN 13748-2:2004:*Terrazzo Tiles – Part 2: Terrazzo Tiles for External Use. EuropeanCommittee for Standardization (CEN), Brussels* , (2004).

[59] Xia, Y., Shi, D., Zhao, R., Yu, K., Liu, M., Mei, H., Xu, L., Zhao, Y., Wang, L. & Yan, J.Iron-rich industrial wasteenhanced low-carbon radiation shielding functional composites. *Journal of Cleaner Production* **449**, 141649 (2024).

[60] Shi, D., Xia, Y., Zhao, Y., Wang, J., Ma, X., Liu, M., Yu, K., Zhang, J., Tian, W. & Zhang, J. Valorization of steel slaginto sustainable high-performance radiation shielding concrete. *Journal of Building Engineering* **91**, 109650 (2024)..

